# Understanding Concomitant Physical and Chemical Transformations of Simvastatin During Dry Ball Milling

**DOI:** 10.1208/s12249-020-01687-z

**Published:** 2020-05-21

**Authors:** Dattatray Modhave, Isha Saraf, Anjali Karn, Amrit Paudel

**Affiliations:** 1 Research Center Pharmaceutical Engineering (RCPE) GmbH, Inffeldgasse 13, 8010 Graz, Austria; 2 Institute for Process and Particle Engineering, Graz University of Technology, Inffeldgasse 13, 8010 Graz, Austria

**Keywords:** solid-state disorder, milling, crystallinity, chemical degradation

## Abstract

The present study investigates concomitant processes of solid-state disordering and oxidation of simvastatin during milling. The separate dry ball milling of crystalline and amorphous powders of simvastatin were conducted at ambient temperature for 10 and 60 min each. The relative crystallinity was determined using X-ray scattering and oxidative degradation was analyzed using liquid chromatography. The physical and chemical transformations in the milled powder were evaluated using modulated differential scanning calorimetry (DSC) and Fourier transform infrared spectroscopy. The disordering during milling of the crystalline powder was found to progressively decrease the crystallinity. For the amorphous starting material, milling for 10 min induced a large extent of recrystallization, while milling for 60 min largely re-amorphized the powder. This solid-state disordering and/or ordering were accompanied by progressive air oxidation during milling. The infrared spectroscopic analysis revealed the molecular manifestations associated with the physicochemical transformations in the disordered solid states. The melting point of simvastatin depressed systematically with the increase in the degree of disorder as well as the degradation. The *in situ* cooling in DSC of milled samples from their molten state led to the formation of the co-amorphous phase between the drug and degradation products, which showed a consistent increase in glass transition temperature with the increase in the content of degradation products. The study overall demonstrates the solid-state re-ordering and disordering of crystalline and amorphous simvastatin accompanied by chemical degradation as the consequence of the mechano-activation.

## INTRODUCTION

Drug substance and drug products are required to remain physically and chemically stable while manufacturing and during shelf-life storage. Any kind of undesired physical or chemical change in the drug substance can seriously impact the quality, safety, and efficacy of the final drug product (1). Understanding the exact causes of instability during product manufacturing and/or storage is essential. During solid product manufacturing, the involved mechanical stress (attrition, pneumatic transport, shear, *etc.*) can (partially) transform crystalline drug powders to an amorphous phase (2). Processes such as milling, sieving, and compaction can induce unintended defects and disorders on the drug crystal surfaces that can potentially trigger physical instability such as polymorphic conversion, crystal dehydration, and salt disproportionation and chemical instability such as drug degradation (3). The milling-induced physical transformation such as mechano-tautomerism, polymorphic conversion, amorphization, and/or chemical degradation is well-documented (4–9). The high energy milling of the powder results in the reduction of crystal size, creating crystal defects and/or active surfaces, and amorphous phases, all of which may depend upon crystal properties as well as milling energy and time (10). Dry ball milling is a widely used process for generating co-amorphous drug delivery systems to enhance bioavailability (11–14). The milling-induced changes in the surface and bulk crystal packing can alter the thermodynamic, kinetic, and mechanical properties (15). One of the consequences can be the initiation of chemical degradation at the mechano-actively disordered/ amorphous surface of the milled crystals. The exact relationships of the type and degree of solid-state disorder to the mechanism and the kinetics of chemical degradation are still not well understood. Moreover, in-process chemical degradation of the amorphous/disordered solid states during milling of chemically unstable drugs has not received rigorous scientific attention.

The statin class of lipid-lowering agents such as lovastatin is known to undergo auto-oxidative degradation in a solid state (16) due to their lower bond dissociation energy (17). Likewise, we reported in our previous study that simvastatin, a widely prescribed lipid-lowering drug (18,19), simultaneously undergoes amorphization and air oxidation during cryogenic ball milling of crystalline state (20,21). As a follow-up, our present work aims towards understanding the impact of room temperature milling of crystalline and amorphous states of simvastatin on the physical and chemical transformation. We hypothesize that amorphous fraction generated at early time points during milling of crystalline simvastatin undergoes mechano-chemical reaction as well as recrystallization in the course of long milling time. To deconvolute these two competing kinetic processes, we compared the evolution of physical and chemical states of simvastatin during milling, starting with amorphous and crystalline powders. In the present study, we also intend to investigate the effect of the oxidative degradation products on the thermo-physical properties of the mechano-activated crystals and amorphous simvastatin. The outcome of the study evidenced the solid-state disordering and re-ordering of crystalline and amorphous forms, respectively, accompanied by chemical degradation during milling of the powder. It was observed that the amorphization during milling can lead to air oxidation. Starting with amorphous material, crystallization dominated during the early phase of milling, while chemical degradation and further amorphization occurred upon longer milling. Subsequently, solidification (after melting) of disordered powders generated co-amorphous systems between simvastatin and its degradation products with higher glass transition temperatures than that of pure drug.

## EXPERIMENTAL

### Materials

Crystalline simvastatin powder was purchased from Shenzhen Nexconn Pharmatechs Ltd., China. The chemicals and reagents were analytical grades and purchased from Sigma Aldrich.

#### Generation of Amorphous Simvastatin Powder *via* Cryo Ball Milling

To generate the amorphous simvastatin powder, the milling of crystalline powder was performed under cryogenic conditions employing liquid nitrogen (21). Using a single stainless steel ball of 2.0 cm in diameter, cryo-milling was carried out at 25 Hz in the Cryogenic Mixer Mill (CryoMill, Retsch Germany). About 2000 mg of simvastatin crystalline powder was transferred in a 50-mL container and subjected to the cryo-milling for a period of 90 min.

#### Dry Ball Milling of Crystalline and Amorphous Powders at Room Temperature

The milling experiments of simvastatin unmilled (crystalline), as well as cryo-milled (amorphous) powders, were performed at ambient temperature using a Retsch Mill (Germany). About 1000 mg of simvastatin powder was milled in a 50-mL container with a single 2.0-cm stainless steel ball at the frequency of 25 Hz. In order to create different degrees of solid disorder, the room temperature milling was carried out for 10 and 60 min in each case. Unmilled (0 min) crystalline and cryo-milled (90 min milled) amorphous samples were considered as a control with 100% and 0% crystallinity, respectively, throughout the study. The different samples are abbreviated as C0, C10, and C60 for unmilled and 10 and 60 min milled, respectively, when crystalline starting material was used. In the case of the amorphous starting material, the samples are designated as A0, A10, and A60 for cryo-milled and 10 and 60 min milled, respectively.

#### Physical and Chemical Characterization of Solid States Generated *via* Milling

Understanding the fundamental solid-state attributes of the pharmaceutical material is important to deconvolute and to postulate the mechanisms of solid-state transformations. During the study, thermo-physical properties, structural factors, and intermolecular interactions of the disordered and chemically impure solid states generated *via* milling were analyzed using thermal, X-ray, and spectroscopic techniques.

#### Differential Scanning Calorimetry

Modulated temperature differential scanning calorimetry (DSC) experiments were performed using the DSC 204 F1 Phoenix (NETZSCH, Germany) to obtain the information about glass transition temperature, melting enthalpy and temperature, and non-isothermal recrystallization temperature and enthalpy (if any). The DSC was equipped with an auto-sampler and intra-cooler (to provide the controlled heating/cooling rate and temperatures). Approximately, 5–10 mg powder mass was used for each sample. The sample was placed in an aluminum pan and covered with a lid with a pinhole and measured in heat-cool-heat mode. During the first step, the sample was heated from 0 to 150°C with a linear heating rate of 5°C/min, an amplitude of modulation of ± 0.5°C, and a modulation period of 40 s. After the first heating step, the sample was cooled to − 10°C at the rate of 10°C/min. In the second heating, similar modulation parameters were used. Temperature and enthalpy calibrations were performed using indium, zinc, and bismuth.

#### Attenuated Total Reflection-Fourier Transform Infrared Spectroscopy

The attenuated total reflection-Fourier transform infrared (ATR-FTIR) spectroscopic analysis was performed to elucidate the change in inter- and intra-molecular interactions such as hydrogen bonding, due to the generation of disordered/amorphous samples and/or degradation impurities. The spectrometer was equipped with a diamond ATR crystal and DLaTGS detector (Bruker Optics, Germany). Initially, the background was recorded without the sample as a blank and then a small quantity of sample was kept on to the ATR crystal. The spectral range of 600 to 4000 cm^**−**1^ and a resolution of 4 cm^**−**1^ were used during measurement. In total, 32 spectral scans were recorded for each measurement.

#### Wide-Angle X-Ray Scattering

The solid-state disorders and the relative degree of crystallinity in the milled samples were further analyzed using wide-angle X-ray scattering (WAXS) (in the range of 17–27° 2*θ*). The WAXS system comprised S3-MICRO, point-focusing, and a detector (Hecus PSD-50, 54 μm/channel); a Cu Kα X-ray micro-source with the instrumental setting of 30 kV/0.4 mA was used in transmission mode. Quartz capillary was used to fill the powder sample. The capillary was kept rotating (0.2 Hz) at room temperature during the measurement of the sample. The total counting time was 1800 s. The Bragg peak positions were used to identify and confirm the particular polymorphic state of the crystalline simvastatin before and after milling. The intensity of the selected Bragg peaks was used for the quantification of the relative degree of crystallinity for each sample (details in the “RESULTS AND DISCUSSION” section) (22).

#### Chemical Characterization of Solid States Generated *via* Milling

##### Ultra-High Pressure Liquid Chromatography

To quantify the chemical degradation generated by milling, the solid samples were dissolved in a selected medium and analyzed using ultra-high pressure liquid chromatography (UPLC). UPLC analysis was performed using a Waters Acquity H-class system having a photodiode array (PDA) detector. A reversed-phase method was developed using a C-18 column and gradient elution mode. For the sample preparation, a combination of acetonitrile and water was used as a diluent in the ratio of 70:30 v/v. The solid powder was dissolved in the dilution solvent to achieve a concentration of 500 μg mL^−1^. Each sample was analyzed in triplicate (*n* = 3). Degradation products were quantified using the relative peak area method. The method details and UPLC and mass spectrometric parameters (used for characterization of degradation products) are stated in our previous study (21).

## RESULTS AND DISCUSSION

### Physical Characterization of the Milled Crystalline and Milled Amorphous Simvastatin Powder

#### Thermal Analysis

**Fig. 1:**
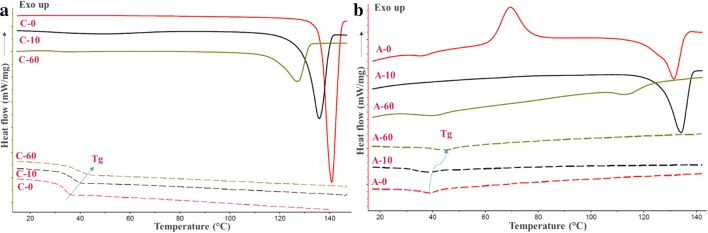
Overlays of DSC thermograms of unmilled (0 min) and milled (10 and 60 min) powders. Left panel (**a**) starting crystalline powder and right panel (**b**) starting amorphous powder. Solid lines represent melting events obtained during the first heating step (in total heat flow) and dashed lines represent glass transition events (in reversing heat flow) obtained during the second heating step (after cooling step). The arrows in thermograms provide the visual guide to the direction of the change in *T*_g_

The thermo-physical properties of solid-state disorders were assessed using mDSC. Using the temperature modulation, the total heat flow was deconvoluted into the heat capacity–related signal (reversing heat flow) and kinetic signal (non-reversing heat flow) and was captured in the heat-cool-heat mode for milled crystalline (Fig. 1a) and milled amorphous samples (Fig. 1b).

DSC measurements were carried out in triplicate for crystalline unmilled and milled samples. The average values of the key thermal transitions are listed in Table I. During the first heating step, the total heat flow for raw crystalline simvastatin (unmilled material) showed the onset of melting (*T*_m_) at 136.7 ± 0.1°C having melting enthalpy (Δ*H*_m_) of 77.4 ± 2.1 J/g. The samples were immediately analyzed after milling by DSC to avoid the variations coming from the sample history like mechanical and molecular relaxation of the disordered phase.

**Table I:** The Values (Average ± Std. Dev.) of the Melting Temperature and Enthalpy and Glass Transition Temperature Obtained During First Heating Cycle and Second Cycle (Acquired During Heating After Cooling The melt) of Milled and Unmilled Simvastatin Samples

Sample name		Crystalline simvastatin			Amorphous simvastatin		
		C0	C10	C60	A0*	A10	A60
Onset of melting in °C (*T*_m_)		136.7 ± 0.1	130.2 ± 0.3	118.3 ± 0.2	127.4 ± 0.1	128.1 ± 0.2	107.1 ± 0.1
melting enthalpy in J/g (Δ*H*_m_)		77.4 ± 2.1	63.1 ± 3.5	27.5 ± 1.7	42.2 ± 3.4	48.4 ± 0.8	6.31 ± 0.1
Glass transition temperature (*T*_g_) in °C	First heating	ND	ND	32.8 ± 0.7	31.7 ± 0.4	ND	32.7 ± 0.7
	Second heating	31.5 ± 1.2	33.7 ± 0.6	35.8 ± 1.0	34.5 ± 0.1	35.1 ± 0.5	40.5 ± 0.1
Relative crystallinity in %		100.0	57.0 ± 1.2	17.7 ± 0.5	0.0	70.6 ± 0.8	5.8 ± 0.1

*The melting event is of the crystals generated during non-isothermal crystallization in DSC (as evidenced by crystallization exotherm, with an onset temperature of 62.5°C and enthalpy of 41.4 ± 1.5 J/g). *ND* not detected

For the 10-min milled sample, *T*_m_ and corresponding Δ*H*_m_ obtained were 130.2 ± 0.3°C and 63.1 ± 3.5 J/g, respectively, while for the 60-min milled sample, the *T*_m_ and the Δ*H*_m_ values further reduced to 118.3 ± 0.2°C and 27.5 ± 1.7 J/g, respectively. The depression in the melting point of the milled powders can be originated from the presence of degradation products and/or due to the formation of (smaller) crystals with surface disorders and imperfections and by the overall decrease in crystallinity. The observed broadening of the melting peaks can either be due to the presence of crystals with defects or the local chemical heterogeneity in the presence of the degraded impurities. Furthermore, the reduction in the melting enthalpy indicates the loss of crystallinity and thus, the generation of an amorphous fraction and imperfect crystals. Besides *T*_m_, the careful inspection of the thermogram obtained during the first heating of the 60-min samples also evidences the presence of a glass transition event with the glass transition temperature (*T*_g_) of 32.8 ± 0.7°C.

Rapid cooling of simvastatin melt samples in DSC with different impurity levels converted to a complete amorphous state as evidenced by a single *T*_g_ event and no melting event was observed in the second heating thermograms. This implies that the cooling rate of 10°C/min was sufficient in the present case to arrest the dynamic disorders of the isotropic melts to convert into glassy amorphous states, irrespective of the level of impurity. As shown in Fig. 1a, a glass transition event was clearly visible for all samples in the reversing heat flow. The *T*_g_ values systematically increased with the increase of the milling time. More precisely, the *T*_g_ values of unmilled, 10-min milled, and 60-min milled samples were 31.5 ± 1.2°C, 33.7 ± 0.6°C, and 35.8 ± 1.0°C, respectively. These observations imply that the oxidative degradation products generated during milling are molecularly mixed with simvastatin during melt solidification and actually anti-plasticize the system. This also means that pure degradation product(s) will have a higher *T*_g_ than simvastatin. The non-isothermal recrystallization exotherm was not seen in any of the cases after the second heating.

Likewise, the amorphous powder generated *via* cryo-milling was also characterized using the same mDSC method and also subjected immediately to RT milling. During the first heating step, the cryo-milled amorphous sample showed a distinct *T*_g_ at 31.7 ± 0.4°C. In addition, a recrystallization exotherm (*T*_c_) and melting endotherm (*T*_m_) were observed with the onset temperatures at 62.5 ± 1.6°C and 127.4 ± 0.1°C, respectively. The RT milled amorphous samples (10 and 60 min) were also analyzed *via* mDSC. As shown in Fig. 1b, for short milling of 10 min, *T*_m_ was noticed at 128.1 ± 0.2°C. However, no distinct *T*_g_ was seen. Whereas for the 60-min milled samples, a *T*_g_ and a *T*_m_ were noticeable at 32.7 ± 0.7°C and 107.1 ± 0.1°C. The presence of trace melting endotherm and higher shift in *T*_m_ during the first heating step suggests the influence of the chemical degradation during long milling run. As seen in the case of A0, the non-isothermal recrystallization upon heating amorphous powder of a low *T*_g_ drug like simvastatin prepared by cryo-milling is reported before (21). The remnant short-range order (of crystal) within amorphous state obtained by mechanically destroying the crystal structure during milling can act as a pre-nucleation cluster during heating of the amorphous phase. Whereas the second heating of amorphous phase obtained by solidification of isotropic melt in DSC lacks recrystallization. In the case of A10, the recrystallization peak was not observed, probably because of either high crystallinity or the rigidity of the remaining amorphous structures or both. Interestingly, recrystallization in DSC was also not seen in case the highly amorphous A60. We hypothesize that this could be probably due to the presence of the higher fraction of the oxidative impurities in the 60-min milled samples, inhibiting the non-isothermal nucleation and crystal growth.

For all the three samples, the second heating (after rapid cooling of melt in DSC) showed a glass transition as the only thermal event. The absence of crystallization and melting peaks demonstrates the formation of complete glassy states. The values of *T*_g_ for the melt-solidified samples progressively increased with the increase in the milling time. For the second heating step, *T*_g_ shifted from initial 34.5 ± 0.1°C to 35.1 ± 0.5°C after the 10-min milling, and even further to 40.5 ± 0.1°C after 60-min milling. We hypothesize that the presence of a higher amount of oxidative impurities in the 60-min milled sample strongly inhibits the crystallization as well as provides the anti-plasticization effect. As described before, like milled crystalline powder, amorphous samples also showed the co-amorphization between drug and degradation products after melt cooling.

The relative percent crystallinity (*X*_c_) for the milled samples was estimated as the ratio of the enthalpy of melting of the crystalline fraction of the milled materials to that of the unmilled crystal (with the assumption of 100% crystallinity). The *X*_c_ values obtained for the milled systems were < 50% for 60 min (C60 and A60) and > 50% for 10 min (C10 & A10). However, this estimation can be affected by the presence of impurities in milled samples. Also, the effect of different melting temperatures observed for milled samples on their melting enthalpy was not incorporated in this simple calculation (the temperature dependency of heat capacity of the crystal). The trends of crystallinity obtained by DSC were overall comparable with the WAXS data (described in the next section).

#### X-Ray Scattering Studies

**Fig. 2:**
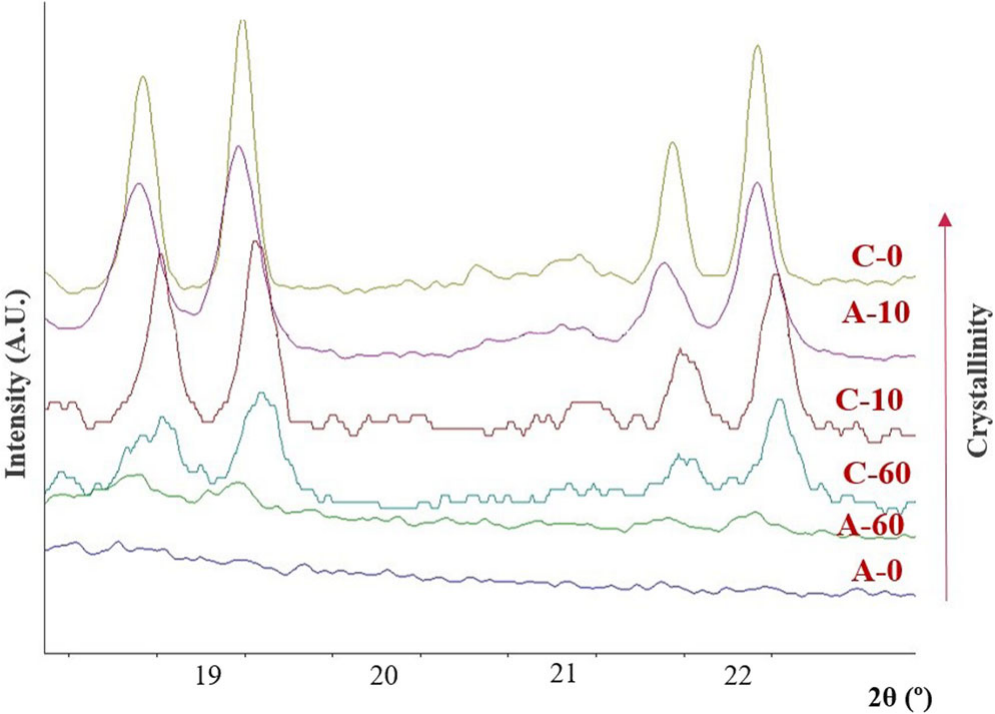
Overlays of WAXS patterns for unmilled and milled samples

The physical changes at the crystallographic scale upon milling the simvastatin crystals were investigated using wide-angle X-ray scattering (WAXS). Milling the crystalline powder led to the progressive decrease in the relative intensity of Bragg peaks, evidencing the loss of crystallinity with the increase in the milling time (Fig. 2). The positions of Bragg peaks remain unchanged upon milling, pointing to the absence of any polymorphic transformation. The WAXS pattern of the cryo-milled sample shows the absence of Bragg peaks suggesting the formation of a fully amorphous state. Upon RT milling of the amorphous samples, the 10-min and 60-min milled samples demonstrated comparable characteristic with the Bragg peak positions. However, the intensity of the Bragg peaks in the case of 60-min milled samples was markedly reduced as compared with the 10-min milled samples (Fig. 2).

For the quantification of the relative crystallinity, the intensity of the Bragg peak at 19.2° 2*θ* in WAXS was used. Simvastatin disorder quantification was reported previously using X-ray diffraction and spectroscopic techniques (22). Upon milling of crystalline samples, the relative crystallinity values for the 10- and 60-min milled samples were found to be 57.0 ± 1.2% and 17.7 ± 0.5%, respectively. The recrystallized amorphous sample showed the relative crystallinity of 70.6 ± 0.8% and 5.8 ± 0.1% for 10 and 60 min, respectively. Overall, the relative crystallinity was in the order of as C0 > A10 > C10 > C60 > A60 > A0 (the trend is in accordance with that obtained using the DSC data).

#### Spectroscopic Evaluation

**Fig. 3:**
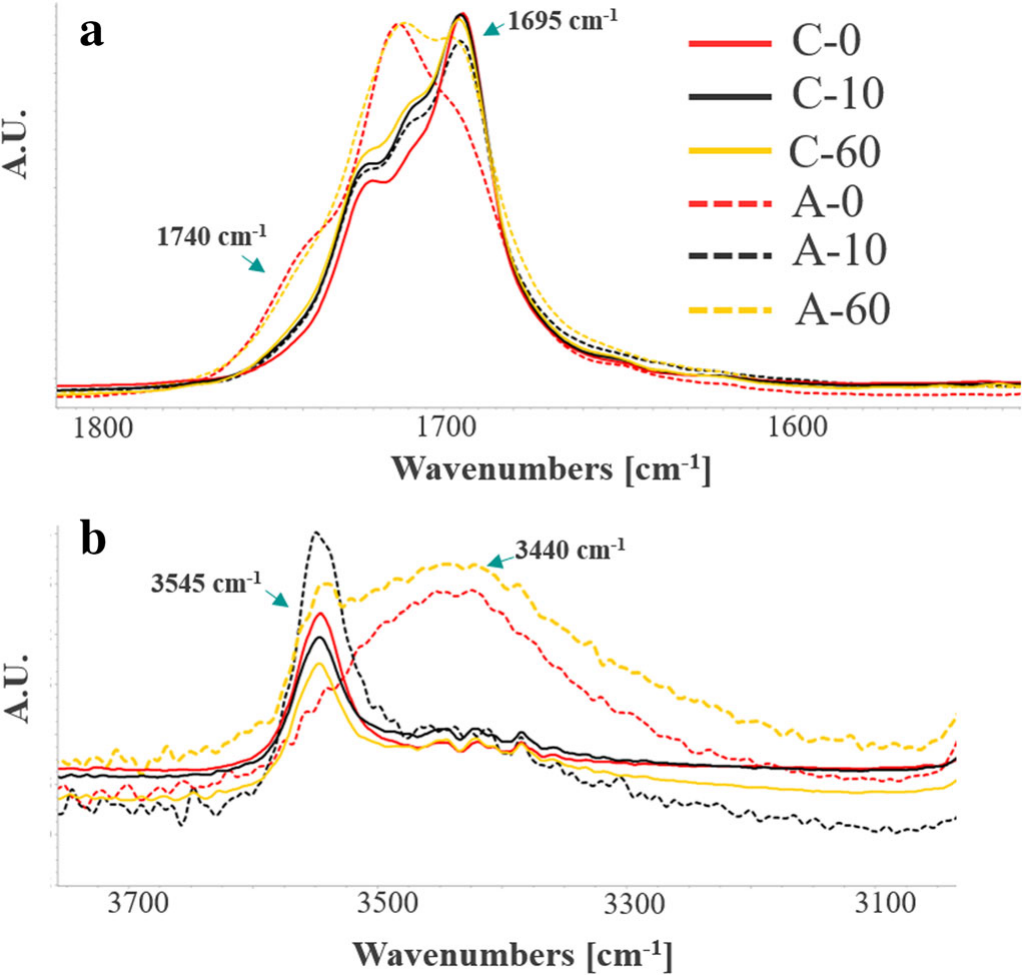
Overlays of partial IR spectra for **a** –C=O region and **b** –O–H vibrational bands, normalized with respect to the most intense peak of the carbonyl region

The intermolecular interactions in different solid states of simvastatin were characterized using ATR-FTIR spectroscopy. The molecular packings in crystals typically constitute the hydrogen bonding, Van der Waals, and electrostatic interactions, which are often disrupted by the generation of crystal disorders. These changes in molecular interactions can result in the broadening of the vibrational bands and a decrease in peak intensity. The comparative partial spectral profiles of amorphous, crystalline, and partially disordered simvastatin are shown in Fig. 3.

For unmilled simvastatin crystal (C0), the C=O region exhibits three mutually overlapping vibrational bands. The most intense peak with the maximum at 1695 cm^−1^ is attributable to the H-bonded C=O of the linear ester. The peaks with the maxima at 1708 and 1720 cm^−1^ could be related to the C=O of free linear ester and closed ester (lactone), respectively. Likewise, in the O-H region of crystalline simvastatin, a characteristic peak with a maximum at 3545 cm^−1^ is noticeable. For the fully amorphous simvastatin obtained *via* cryo-milling (A0), the notable broadening of the vibrational bands occurred in the region of 1750–1710 cm^−1^ (Fig. 3a). More precisely, the relative intensity of H-bonded C=O of linear ester decreased with a distinct increase in the intensity of non-H-bonded C=O of the same functional group. In addition, a unique shoulder appeared towards a higher wavenumber with a peak maximum at 1740 cm^−1^. This could probably be attributable to a specific non-H-bonded C=O species that is only present in the amorphous state. These observations point to the disruption of the intermolecular H-bonds upon amorphization induced by milling. The O-H region (Fig. 3b) of the fully amorphous powder (A0) lacks the crystalline peak with the maximum at 3545 cm^−1^. Instead, a broad peak with the maximum at 3440 cm^−1^ appeared.

Upon milling, the characteristic spectral changes were noticeable. In the samples generated by milling crystalline simvastatin, a systematic broadening of vibrational bands in C=O regions was noticed. Concurrently, the intensity of the OH peak with the maximum at 3545 cm^−1^ decreased from C0 to C60. The milled samples generated from the amorphous starting material (A10 and A60) also showed unique spectral changes over milling time. Specifically, the peak with a maximum at 1695 cm^−1^ decreased after 60-min milling (A60). This was accompanied by an appearance of a broad peak with the maximum at 1740 cm^−1^, like in the case of A0. In the O-H region, with increasing amorphous fraction, the diffuse peak (3440 cm^−1^) was increasingly visible. Interestingly, for A60 which shows a trace crystallinity (see DSC and WAXS sections), a trace of the sharp crystalline vibrational band (3545 cm^−1^) was still notable confirming the residual crystallinity for A60. On the other hand, the A10 showed a comparable spectral profile as that of the crystalline sample (C0).

**Fig. 4:**
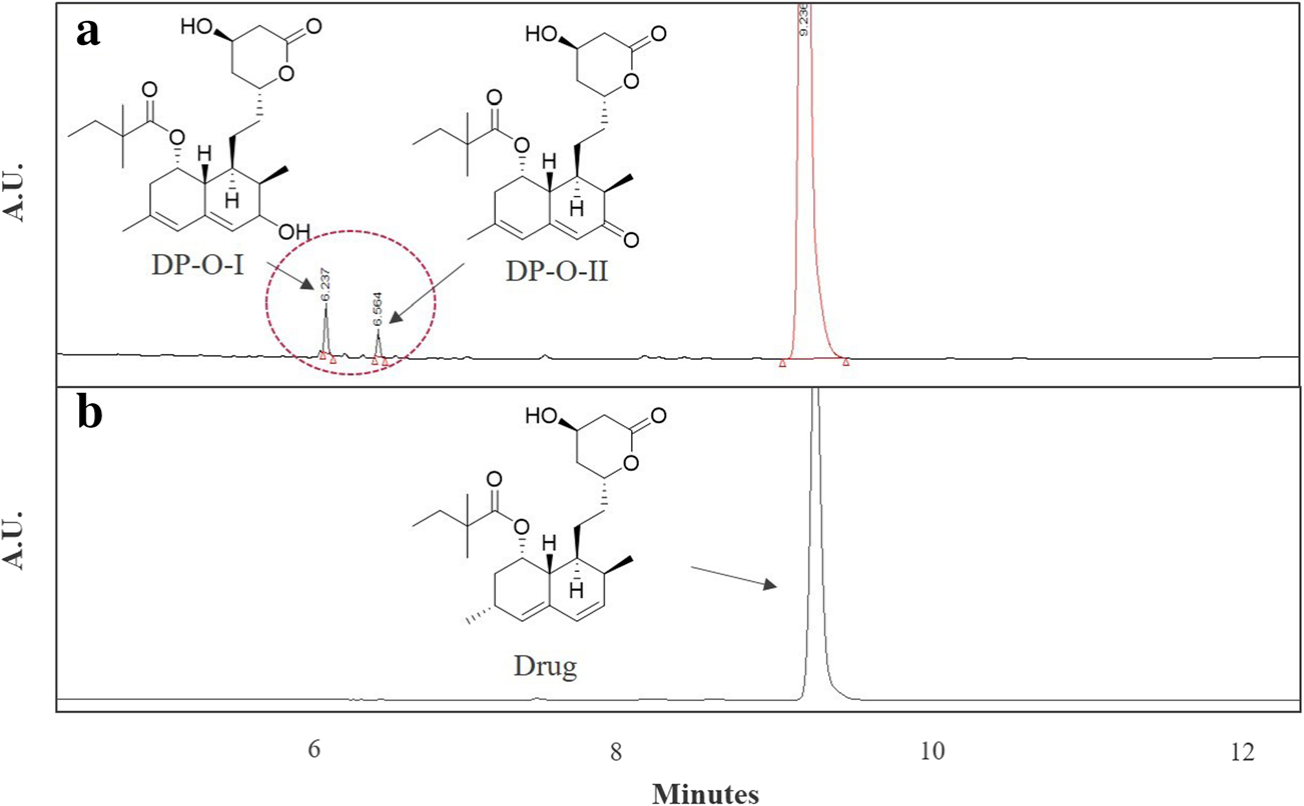
Representative chromatogram of **a** sample showing drug peak (simvastatin) and oxidative degrades (DP-O-I and DP-O-II) and **b** unmilled simvastatin (corresponding chemical structures are in the inset)

Milling-induced physical changes (solid-state disordering and ordering) indeed appear to accompany the chemical degradation (keto and enol product formation) (Fig. 4). As the amorphization and degradation can occur partly together during milling, this could have an impact on the characteristic spectral changes. The tendency of shouldering towards the higher wavenumber (1740 cm^−1^) in the C=O region follows the order of amorphicity, which supports the results observed *via* WAXS. Overall, the rest of the spectral regions of the partially disordered samples obtained showed the combined features of the fully amorphous and the fully crystalline sample. In addition, the influence of chemical changes induced by milling on the spectral profiles was distinct as well. For example, in the predominantly amorphous A60 sample where the highest degradation was also observed (refer to the “Ultra-High Pressure Liquid Chromatography” for the degradation content), the relative intensity of 1695-cm^−1^ peak was towards that of the fully crystalline sample. This contradicts its physical state (if it were in the pure state). Therefore, we speculate that this elevated intensity of C=O (in comparison with the fully amorphous sample) could be presumably due to the presence of additional C=O in the naphthyl ring of keto degradation product (Fig. 3a). Likewise, the OH of enol degradation product formed in A60 could contribute the broad peak appearing at 3440 cm^−1^, in addition to its higher amorphicity. Full confirmation of these spectral interpretations will require the analysis of the impurity samples alone in their crystalline and amorphous states.

### The Extent and Type of Chemical Degradation Products Generated during Milling

The chromatographic data show the formation of two main polar degradation products for the milled samples that were eluting prior to the drug peak and were absent in an unmilled sample (Fig. 4). The total oxidative impurities (mean ± SD, *n* = 3) increased from 0.3 ± 0.03% for 10-min milled sample to 1.9 ± 0.05% for a 60-min milled sample, upon milling crystalline simvastatin. The degradation products (DPs) with RRT 0.68 (DP-O-I, enol-simvastatin) and RRT 0.71 (DP-O-II, keto-simvastatin) are shown in Fig. 4. The detailed structural elucidation of these oxidative products was made in our earlier study (21).

For the RT milled amorphous samples, the types of degradation products (DP-O-I, and DP-O-II) were identical to that of RT milled crystalline samples. In the beginning, during cryo-milling (while complete amorphization of crystalline powder takes place), oxidative impurities were observed to rise to the level of 0.67 ± 0.01%. This could be due to the participation of oxygen during low-temperature milling, signifying susceptibility of disordered amorphous fractions towards surrounding oxygen. After milling the amorphous simvastatin for 10 min, the increase in the level of the total impurities (0.74 ± 0.01%) was minimal. Notably, the oxidative product increased prominently to 6.09 ± 0.23% upon milling the amorphous sample at RT for 60 min. In connection with the physical transformation discussed above, it is apparent that milling amorphous samples up to 10 min at RT leads to crystallization while chemical transformation remains minimal. Whereas milling progression to 60 min induces sufficient mechano-activation of the surface energy of the powder where amorphization of the generated crystals and chemical degradation of the amorphous fraction take place simultaneously. In future studies, it would be interesting to either sample at the shorter intervals and analyze physical and chemical transformation or deploy *in situ* techniques that can monitor the change occurring during milling. The data from such a study can provide a crucial insight into the threshold mechano-activation for triggering physical and chemical transformation while powder energy evolves during milling. This has serious industrial implication in designing a dry milling process using material science principles. On the other hand, this can enable developing RT ball milling as a rapid approach of assessing the physical and chemical transformation of pharmaceutical solids considering their mutual impact on the kinetics and mechanisms.

### Evaluation of the Milling-Induced Physical and Chemical Transformations

**Fig. 5:**
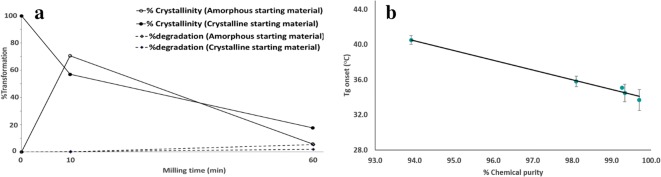
**a** The plots of physical transformation (amorphization and/or crystallization) (circles and solid lines) and chemical degradation (diamond and dashed lines) as a function of milling of amorphous (open symbols) and crystalline (filled symbols) simvastatin. **b** The plot of glass transition temperature (*T*_g_) obtained in the second heating curves of DSC signals as a function of the chemical purity of different simvastatin samples. The solid line is the linear fit to the experimental data (correlation coefficient 0.97, slope − 1.16, and intercept 2.56)

Figure 5a depicts a path of physical and chemical transformation of simvastatin occurring in the course of room temperature ball milling of amorphous and crystalline starting materials. Within 10 min of milling, the extents of chemical transformation are comparable for both amorphous and crystalline materials (degradation < 1%). Within this period of milling, starting with amorphous and crystalline simvastatin showed opposite trends of physical transformation. More precisely, mechano-activation results in the amorphization of crystalline powder while the amorphous powder undergoes mechano-activated crystallization. We measured the powder temperature immediately after opening the milling jar using thermocouple and the average value was found to reach up to approx. 55°C (nearly *T*_g_ + 25°C) during room temperature milling. Therefore, the transient collision and friction of powder surfaces while colliding within themselves and with the milling ball surface and the wall of the containers can induce both ballistic process of crystal amorphization and thermodynamically driven crystallization of amorphous fraction present in its super-cooled state (23). Therefore, a higher relative crystallinity obtained by milling amorphous material compared with that obtained from crystalline material after 10 min appears to be due to the higher speed of crystallization compared with amorphization induced by mechano-activation. Also, mechanical properties such as hardness and thermal conductivity (and heat capacity) of the crystalline material which could also contribute to higher crystallinity. From 10 to 60 min, while the amorphization continues for the crystalline starting material, amorphous starting material, which otherwise crystallized until 10-min milling, also now largely transforms into the amorphous state. This implies that both the starting materials possibly attain similar energetic states with closer degrees of crystallinity within 10 min. More precisely, in the case of amorphous starting material, the remaining amorphous fraction might have so relaxed that they can no longer cross the activation energy of mechano-activated nucleation and crystal growth. Rather, the fraction crystallized during milling amorphous powder for 10 min starts following the similar path of physical transformation as of crystalline starting material upon continuing milling further. From a chemical transformation standpoint also, milling for 10 min seems to generate the configurations of molecules on the amorphous/disordered powder surface that can now readily cross the activation energy barrier of oxidation upon further mechano-chemical energy input. This could have possibly triggered degradation while milling from 10 to 60 min.

To our knowledge, this is one of the first reports of mechano-active treatment of fully amorphous API. Starting with crystalline API, it is expected that the amorphous fraction generated during early time points of milling will experience mechano-chemical stress throughout the process. Therefore, our intention of milling amorphous state of API was to deconvolute the physical and chemical transformation it undergoes during the process of milling. Here, the amorphous starting sample undergoes significantly higher degradation than that of the crystalline counterpart after 60 min, despite the crystallinity at the preceding time point (10 min) being slightly higher. This points to the possibility of the higher degree of dispersity and the smaller size of the crystallized fraction generated after milling the amorphous sample for 10 min. If so, this could provide a higher effective surface for degradation to occur upon further mechano-activation, despite the comparable crystallinity with C10. As no clear *T*_g_ is noticeable for the latter sample, the disorder generated could be the lattice defects and surface imperfections (pre-amorphous disorder), which could be relatively less active than the amorphous portion remaining in A10. Also, this could have partly contributed to a slightly higher extent of amorphization of A60 compared with that of C60. Shamblin *et al*. reported the relationship between chemical reactivity and structural relaxation times in pharmaceutical glasses (24). In this context, the structure-energetics relationship of simvastatin is reported in the literature (25). In general, the disordered particles generated during milling are assumed to constitute imperfect crystal core covered by the amorphous shell (26). The appearance of clearly visible *T*_g_ and the reduction of melting enthalpy after 60-min milling indicate that the particles in milled powder are rich in amorphous disorder, in addition to the core crystals with defects. Considering the present data are limited to a single intermediate time point, many speculations made above will again require further thorough investigations. For example, it will be worthwhile to follow both physical and chemical transformations occurring during milling of the two solid states using operando spectroscopy and/or *in situ* (synchrotron) X-ray scattering studies that can register an entire profile of transformation. Such a study can involve other statin congeners so that a deeper knowledge of structure-processability-property relationships can be established, which helps deriving kinetic models for concerted amorphization and degradation.

Melt cooling of the samples with different purities was performed with an intention to understand the impact of structurally similar impurities on the amorphous glass of the drug. A single *T*_g_ of the co-amorphous drug and impurities observed in the second heating cycle implies that the oxidative degradation products generated by milling are molecularly miscible with simvastatin. The presence of hydroxyl and keto groups in the chemical structures of drug and degradation products indeed suggests a strong possibility for the formation of the co-amorphous system through hetero-molecular hydrogen bonding. The linear increase in mixed-phase *T*_g_ obtained in second heating as a function of impurity content implies the anti-plasticizing effect of degradation products (Fig. 5b). Long ago, Elder reported a similar observation for the melt cooled samples of the structurally similar lovastatin (16). It is noteworthy that the extent of change of *T*_g_ as a function of the change in purity for lovastatin is around 10 times lower than that for simvastatin (comparing the slopes of the present study *versus* that of Elder). This implies that despite being the congeners, the extent of physical interactions of degradation products with simvastatin and lovastatin markedly differs. More precisely, the strength of anti-plasticization is much higher in the case of simvastatin. Looking closer to the first and second heating DSC signals of C60, the *T*_g_ in the first heating run is closer to that of fully amorphous pure simvastatin. Thus, it might be the case that amorphous simvastatin generated during milling is not intimately mixed with the degradation products generated as the degradation products probably are either crystalline or are in a separate amorphous state. In terms of further industrial relevance, the *T*_g_ of the amorphous system of the drugs like simvastatin can increase for the product formed *via* thermal processing like melt extrusion, if degradation products are formed. Therefore, such understanding is important to justify the unexpected variability of physical properties like *T*_g_ originating from the presence of degradation-induced impurities, which subsequently can alter the stability of the amorphous phase. Overall, this study portrays the inter-relationship of the physical and chemical properties of the amorphous pharmaceutical solid.

The abovementioned milling-induced physical and chemical transformations are based on the analysis of the powder samples immediately after milling. Considering that the storage of the product before administration to the patient impacts subsequent transitions, it becomes crucial to also temporally follow and evaluate molecular relaxation kinetics of mechano-activated solids. It will be worth investigating the relation of the various solid-state properties and purity to the solubility and bioavailability of the drugs.

### Relationship Between Melting Parameters and Chemical Purity

**Fig. 6:**
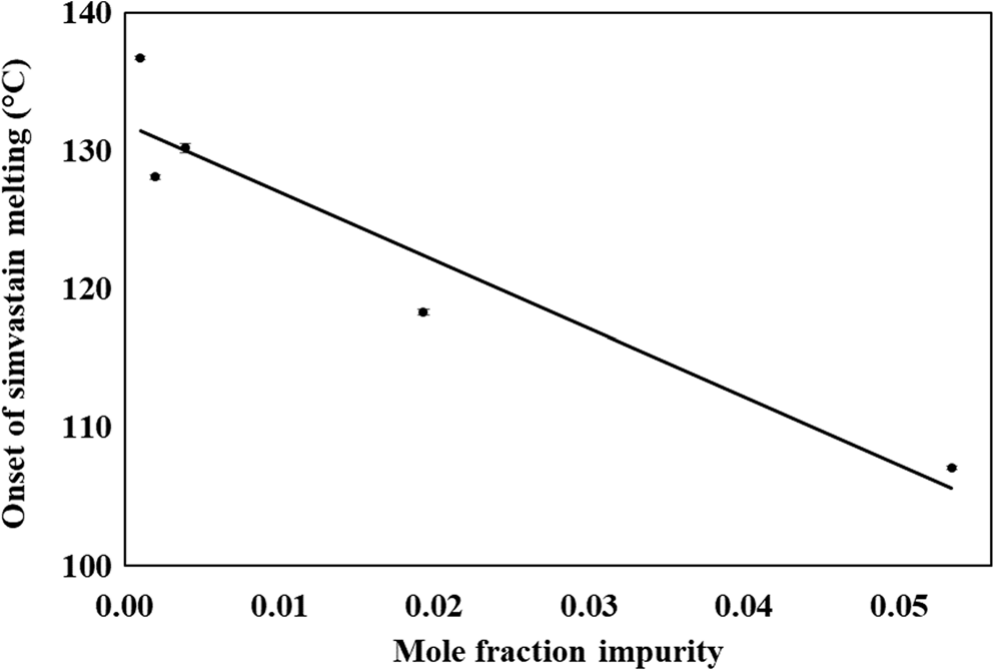
The plot of the onset temperature of simvastatin melting *versus* the mole fraction oxidative impurity content in the milled samples

The relationship between melting point and impurity content is well-known. The van’t Hoff equation relates the extent of the depression of the melting point of a crystalline material as a function of the quantity of the impurity present, as presented below:

$$ \left(\ {T}_0-{T}_S\right)=\frac{R{T_0}^2\ X}{{\varDelta H}_{\mathrm{S}}} $$

where *T*_S_ and Δ*H*_S_ are the observed melting temperature (in °C) and the heat of fusion (in J/g) of the sample containing *X* mole fraction impurity, *T*_0_ is the melting temperature of pure reference, and *R* is the gas constant. The presence of impurity decreases the chemical potential of the system and thereby reduces the temperature of solid-liquid equilibrium, which is in fact the melting onset of the given crystalline material. The plot of the melting onset of simvastatin (determined *via* DSC) as a function of impurity content in the sample generated *via* milling (measured *via* UPLC) depicted in Fig. 6 shows a linear relation. With the presence of the impurity, the melting enthalpy of the main component is also expected to decrease systematically. Such a trend can be clearly visible if there is no change in the physical state (such as polymorphic form and amorphous content) of the main component and the only variable is the change in the impurity content. In our present case, together with the increase in the impurity, milling also leads to a decrease in the crystalline content of the simvastatin which further contributes to the decrease in the heat of fusion. Therefore, the estimation of the impurity content by analyzing the thermal data using van’t Hoff equation is not straight-forward here.

## CONCLUSION

Through this study, we were able to demonstrate concomitant physical and chemical transformation that can occur during the drug substance/product manufacturing process involving mechano-activation such as milling. Solid-state disorders were generated by mechano-activation of crystalline and amorphous simvastatin powder *via* dry ball milling. The evolution of thermo-mechanical energy during milling was found to generate amorphous fraction and other types of crystalline disorders and, at the same time, transiently reactive environment for air oxidation of simvastatin. This case study also illustrates the application of DSC and FTIR spectroscopy as a rapid and inexpensive screening tool for the simultaneous determination of physical (crystal defects, partially crystalline, and amorphous states) and chemical (drug degradation) transformation, applicable during API processing like milling and crystallization*.* In addition to the signature of amorphization, glass transition temperature (*T*_g_) also was found to serve as an important descriptor of chemical change occurring during solid-state disordering. Overall, the results of this study bring further knowledge of the concerted phenomena of solid-state disordering and chemical degradation induced *via* mechano-activation. These aspects are directly relevant to the processing, quality, and stability of pharmaceutical solids.
